# Cultural engagement and mental health: Does socio-economic status explain the association?

**DOI:** 10.1016/j.socscimed.2019.112425

**Published:** 2019-09

**Authors:** Daisy Fancourt, Andrew Steptoe

**Affiliations:** Department of Behavioural Science and Health, University College London, UK

**Keywords:** Cultural engagement, Mental health, Depression, Socio-economic status, Social gradient

## Abstract

There is a growing body of literature suggesting that the arts can support mental health. However, both arts participation and cultural engagement are unevenly patterned across the population, with a strong social gradient. This social gradient is also evident in mental health. So it remains unclear whether the relationship between arts engagement and mental health can in fact be explained by socio-economic status (SES). This study explores this question specifically in relation to cultural engagement (e.g. visiting museums/galleries/cinema/theatre/concerts) using data from 8780 adults aged 50 + from the English Longitudinal Study of Ageing. We used a statistical triangulation approach, running three separate sets of analyses that each have different strengths and address different statistical limitations or biases. Using logistic regression, the relationship between cultural engagement and mental health was still present when including covariates relating to SES, and there was no evidence of moderation by SES either through the inclusion of interaction terms or stratification. Using propensity score matching, matching participants based on their SES, we also consistently found evidence of the relationship. Finally, using fixed-effects regression which takes account of all time-invariant factors (which include multiple aspects of SES) even if unobserved, we also found no attenuation of the relationship. Overall, this confirms previous reports that cultural engagement is linked with a lower odds of depression amongst adults aged 50 + by demonstrating a robust association in a nationally-representative sample of older adults. While SES does explain around half of the association between cultural engagement and depression, we found no evidence that it either acts as a moderator or the main explanatory factor, with independent associations maintained across all three approaches. However, the fact that higher SES is associated with more frequent engagement indicates that, in population terms, SES is still an important determinant of the salutogenic impact of culture.

## Highlights

• Cultural engagement is related to mental health independent of socio-economic status.
• Socio-economic status (SES) explains only half of the association.
• SES does not act as a moderator of the relationship.
• The relationship remains when matching participants on SES.
• The relationship remains when accounting for all unidentified time-constant factors.

## 1 Introduction

There is a growing body of literature suggesting that the arts can support mental health. Both observational and intervention studies have found protective associations or effects between arts participation (e.g. actively engaging in arts activities such as singing or dancing) or cultural engagement (e.g. visiting museums, the theatre or concerts) and positive wellbeing (Daykin et al., 2018; Fancourt and Steptoe, 2018a; Osgood et al., 1990), the prevention of mental illness (Cohen et al., 2006; Coulton et al., 2015; Fancourt and Steptoe, 2018; Fancourt and Tymoszuk, 2018; Jones et al., 2012), and the management or treatment of mental health conditions (Fancourt et al., 2016a, 2016b; Fancourt and Perkins, 2018; Mössler et al., 1996). Mechanistically, this relationship appears to exist through a combination of psychological, social and behavioural factors. For example, cultural engagement can support emotion regulation (including distraction, problem solving and the building of self esteem) (Fancourt et al., 2019; Hoffmann and Russ, 2012; Ivcevic and Brackett, 2015), provide protective cognitive stimulation (Camic and Chatterjee, 2013; Wang and Blazer, 2015), provide social interaction which can be a source of social support as well as buffer stress (Cohen, 2006; Cohen and Wills, 1985), reduce sedentary behaviours associated with depression (Teychenne et al., 2010), and support coping skills (Hutchinson et al., 2003; Perkins et al., 2018, 2016).

However, there is a well-known social gradient in cultural engagement (Alderson et al., 2007; Reeves and Vries, 2016), with cultural and economic positions intimately connected (Le Roux et al., 2008). This gradient appears to be particularly strong for cultural engagement compared with arts participation, for which only education appears to be a consistent predictor (Reeves, 2015; Yaish and Katz-Gerro, 2012). This gradient is thought to be in part due to logistical factors, such as higher levels of disposable income and closer proximity to cultural venues; in part due to socialisation, with those from higher socio-economic status (SES) backgrounds more likely to be ‘exposed’ to cultural activities, especially cultural engagement (Reeves and Vries, 2016); and potentially in small part due to higher levels of information processing capacity amongst those of higher educational attainment (Reeves and Vries, 2016). Indeed, it has even been proposed that cultural engagement may be related to health inequalities and may lead to increased socio-economic inequalities (Lamont et al., 2014).

This gradient in cultural engagement occurs in parallel to a social gradient in mental illness. Risk factors for many common mental disorders are heavily associated with social inequalities, with disproportionately high risks amongst those from lower socio-economic status backgrounds (World Health Organization, 2014). The mechanisms underlying this association relate to political, social, economic and environmental factors, including differences in access to natural and built environment (such as access to community spaces including green space), and cultural and social norms (Allen et al., 2014).

Given, therefore, that there are evident social gradients across both cultural engagement and mental health, it is important to ascertain whether cultural engagement and mental illness are independently related or whether they are in fact explained by SES. Given that all specific statistical tests contain biases, many of which could affect the exploration of this question, this paper utilises a statistical triangulation approach, applying three contrasting statistical approaches to consider the consistency of results: (i) adjustment, moderation and stratification using logistic regression models, (ii) propensity score matching, and (iii) fixed-effects regression analyses.

## 2 Methods

### 2.1 Participants

Participants were drawn from English Longitudinal Study of Ageing (ELSA): a large, longitudinal cohort study representative of the English population of people aged ≥50 years established in 2002 (Steptoe et al., 2013). The study received ethical approval from the National Research Ethics Service and all participants gave informed consent. We specifically worked with data from Wave 2 (2004/05) across every biennial wave through to Wave 8 (2016/2017); a total of 7 waves and 12 years of data. This provided a core sample of 8780 participants, all of whom were included in analyses.

### 2.2 Measures

#### 2.2.1 Cultural engagement

Cultural engagement was measured via self-report, asking participants about the frequency with which they currently visit (1) the theatre, concert or opera, (2) the cinema, and (3) an art gallery, exhibition or museum. Frequencies for each of the three activities were measured as never, less than once a year, once or twice a year, every few months, about once a month, or twice a month or more. Responses were combined to create an overall index of how frequently participants did any of these activities. For our main analyses, this was then recoded as infrequent (never, less than once a year, or once or twice a year) vs frequent (every few months, about once a month, or twice a month or more). Our sensitivity analyses tested alternative cut-offs.

### 2.3 Depression

Depression was measured using the 8-item Centre for Epidemiologic Studies Depression Scale (CES-D) (Radloff, 1977). This assesses negative affect symptoms or somatic complaints experienced in the past week using a binary reporting scale, with the total number of symptoms summed (0–8) and a score of 3 or greater denoting the presence of depression (Turvey et al., 1999; White, 2015). To identify whether participants scored above the threshold for depression at any of the waves across the 12 years, we assessed their overall CES-D score at all waves and if a score was 3 or greater at any wave, they were classed as having experienced depression.

### 2.4 Socio-economic status

Socio-economic status was assessed with net non-pension wealth quintiles, highest educational attainment (no qualifications; educational qualifications at age 16; educational qualifications at age 18; further educational qualifications), occupational status (using the three-point National Statistics Socio-Economic Classification (NS-SEC) which categorises individuals into routine/manual occupations, intermediate occupations, and professional/managerial occupations), and employment status (full-time; part-time; not in employment). We additionally included age (continuous) and gender (reference male) as covariates.

### 2.5 Statistics

#### 2.5.1 Study 1

We used logistic regression analyses to explore whether cultural engagement was associated with a lower odds of experiencing depression over a 12 year period. For these analyses, cultural engagement and covariates were measured at baseline and depression was measured at every wave from baseline across the 12 years (7 waves). We ran five sets of analyses: (i) the first adjusted just for age and gender, (ii) the second adjusted additionally for all identified socio-economic factors, (iii) the third additionally adjusted for baseline depressive symptoms, (iv) the fourth adjusted for age, gender and all identified socio-economic factors, but only included participants who were free from depression at baseline, (v) the fifth explored whether SES was a moderator by including an interaction term between cultural engagement and wealth, education and social status (all moderators simultaneously entered into the model) and then stratified based first on wealth (lowest two quintiles vs highest three quintiles), second on education (no qualifications vs qualifications at age 16 or above), and third on social status (routine/manual, intermediate or managerial/professional). Our analyses met all regression assumptions. Differences in the size of odds ratios (ORs) between nested models were computed as (OR (E + C) – OR (E))/(1 - OR (E)) * 100, where OR = odds ratio, E = exposure, and C = covariates.

Approximately 30.5% of participants were missing data on one variable or more. To deal with missing data, we used multiple imputation using chained equations, which included all SES along with age and gender variables in the prediction model to generate 50 imputed datasets (each had a final n = 8780). The missing-at-random assumption was strengthened by the fact that some of the same variables used to predict cultural engagement are also known to predict non-response in ELSA (including age, education and wealth) (Steptoe et al., 2013). Analyses on the core un-imputed dataset produced comparable results so we used the imputed data set for greater statistical power.

#### 2.5.2 Study 2

We used propensity matching to create a cohort of individuals who infrequently engaged in culture and individuals who engaged regularly in culture, matched on socio-economic variables. For these analyses, cultural engagement and covariates were measured at baseline and depression was measured at every wave from baseline across the 12 years (7 waves). We calculated the propensity score (logit model) for each individual based on age, gender, employment status, education, wealth and occupational status, and then used nearest available Mahalanobis metric 1-to-1 matching method without replacement, using a caliper size of 0.001 using the Stata module *psmatch2* (Leuven and Sianesi, 2018). Success of the propensity score matching was assessed using Rubin's B < 25 (B = 10.4), Rubin's R of 0.5–2 (R = 1.09) and a percentage bias of <10% for each covariate (bias = 0.6–7.2%) (Morgan, 2018; Rubin, 2001). To compare the prevalence of depression between matched pairs, we calculated odds ratios and exact confidence intervals for matched case-control data using *mcc* (Miettinen, 1976). We ran two sets of analyses: (i) a comparison between matched pairs of whether participants had depression at baseline, (ii) a comparison between matched pairs as to whether they experienced depression at any point over the 12 year follow-up including at baseline. For these analyses, cultural engagement and covariates were measured at baseline and depression was measured at every wave from baseline across the 12 years (7 waves).

#### 2.5.3 Study 3

We used fixed-effects regression to estimate the time-varying relationship between cultural engagement and depression while accounting for time-variant and time-invariant factors. For these analyses, cultural engagement, covariates and depression was measured at every wave from baseline across the 12 years (7 waves). Fixed-effects regression explores within-person variation with individuals serving as their own reference point, compared with themselves over time. So all time-invariant covariates (including many markers of socio-economic status), are accounted for, even if unobserved (Allison, 2009). Data were strongly balanced. We used a Hausman test to confirm the selection of a fixed effects over a random effects model. The modified Wald test for group-wise heteroscedasticity was significant so we applied sandwich estimators. We also tested if coefficients for all years were jointly equal to zero, but as they were not, we included time-fixed effects in our model. We ran two sets of analyses: (i) we assumed that demographics (such as gender) and markers of socio-economic status (such as social class, employment status, educational attainment and wealth) were time-invariant (so included in the model automatically, even if unobserved), (ii) we re-ran the model assuming that wealth and employment status were in fact time-variant and therefore modelled across each wave. As in Study 1, we used multiple imputation. However, analyses on the core un-imputed dataset produced comparable results.

Sensitivity analyses for all three studies tested the cut-off of cultural engagement. We re-categorised our binary index as “never engaging” vs “engaging at any level of frequency” and repeated the analyses of the three studies above. Results are shown as supplementary figures and tables. All analyses were carried out using Stata v14 (StataCorp, College Station, TX).

## 3 Results

### 3.1 Study 1

There was evidence of a distinct social gradient across infrequent vs frequent cultural engagement, with those who engaged more frequently more likely to be better educated, in work, of higher wealth and with a history of higher occupational status, as well as being slightly more likely to be female (see Table 1).

**Table 1:** Demographic and socio-economic factors by frequency of cultural engagement for all respondents [infrequent vs frequent cultural engagement].

	Unmatched data (Study 1 & Study 3) N = 8780		p
	Infrequent cultural engagement (N = 4375)	Frequent cultural engagement (N = 4405)	
Age, mean (SD)	67.0 (10.10)	66.9 (10.1)	.44
Female, %	52.2%	57.8%	**<.001**
Education, %			**.001**
No qualification	49.7%	38.8%	
Qualification at age 16/GCE/O level	16.5%	16.8%	
Qualification at age 18/A level	27.1%	27.1%	
Degree/further qualification	6.8%	17.3%	
Working part-/full-time, %	29.5%	33.9%	**<.001**
Wealth quintile, %			**<.001**
Lowest	22.8%	17.2%	
2nd	21.8%	18.2%	
3rd	21.4%	18.7%	
4th	19.0%	20.9%	
Highest	15.0%	25.0%	
Occupational status across lifespan, %			**<.001**
Managerial/professional occupations	25.6%	36.2%	
Intermediate occupations	24.8%	24.8%	
Routine/manual occupations	49.6%	39.1%	

When controlling just for age and gender, cultural engagement was associated with a 48% lower odds of experiencing depression over a 12 year period (Table 2, analysis i). SES variables accounted for 48% of this association (Table 2, analysis ii). Nevertheless, when controlling for SES variables, the relationship between cultural engagement and depression remained significant, with cultural engagement still associated with a 25% lower odds of experiencing depression over a 12 year period (Table 2, analysis ii). When additionally controlling for depressive symptoms at baseline, there was still a 15% lower odds of developing depression over the 12 year follow-up (Table 2, analysis iii). When restricting the sample to those without depression at baseline, there was a 19% lower odds of developing depression over the 12 year follow-up (Table 2, analysis iv).

**Table 2:** Odds ratios for experiencing depression over a 12 year period: results from logistic regression analyses [infrequent vs frequent cultural engagement].

	OR	95% CI	p
(i) Odds of experiencing depression over 12 years adjusted only for age and gender	0.52	0.46–0.58	**<.001**
(ii) Odds of experiencing depression over 12 years adjusted for age, gender and SES	0.75	0.66–0.85	**<.001**
(iii) Odds of experiencing depression over 12 years adjusted for age, gender, SES & baseline depression	0.85	0.75–0.98	**.026**
(iv) Adjusted odds of developing depression over 12 years if free from depression at baseline	0.81	0.69–0.95	**.008**
(v) Odds of experiencing depression over 12 years stratified by SES			
Lowest two wealth quintiles (n = 3497)	0.68	0.56–0.84	**<.001**
Highest three wealth quintiles (n = 5265)	0.76	0.65–0.89	**<.001**
No educational qualifications (m = 3880)	0.77	0.62–0.96	**.021**
Educational qualifications at age 16 or above (n = 4893)	0.71	0.61–0.82	**<.001**
Routine/manual occupational status (n = 3894)	0.74	0.61–0.92	**.005**
Intermediate or managerial/professional occupation status (n = 4857)	0.75	0.64–0.88	**<.001**

There was no evidence that wealth, education or occupational status were moderators of this relationship (none of the interaction terms was significant for any level of the SES variables). Further, the relationship was found consistently amongst those of lower wealth, higher wealth, with no educational qualifications, with educational qualifications at age 16 or above, of routine/manual occupational status, or of intermediate, managerial or professional occupational status (Table 2 v). When repeating analyses using an alternative threshold (never vs any engagement) all results remained (see supplementary material).

### 3.2 Study 2

**Fig. 1:**
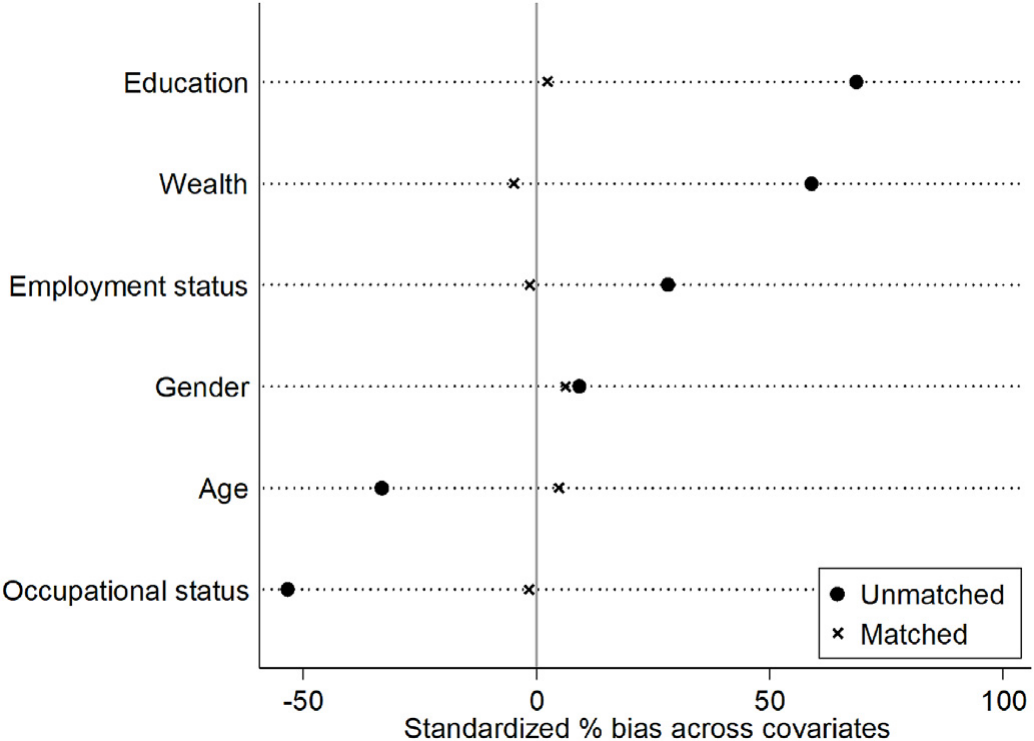
Percentage bias across demographics and SES before and after matching [infrequent vs frequent cultural engagement].

When matching participants based on age, gender and SES, statistical differences between those who engaged infrequently vs frequently in cultural engagement were removed (see Table 3 & Fig. 1).

**Table 3:** Demographic and socio-economic factors by frequency of cultural engagement for matched pairs of respondents [infrequent vs frequent cultural engagement].

	Matched data (Study 2) (N = 4006)		p
	Infrequent cultural engagement (N = 2003)	Frequent cultural engagement (N = 2003)	
Age, mean (SD)	64.7 (9.0)	65.2 (8.6)	.12
Female, %	56.2%	59.3%	.051
Education, %			.47
No qualification	30.0%	26.9%	
Qualification at age 16/GCE/O level	18.7%	24.4%	
Qualification at age 18/A level	38.8%	34.4%	
Degree/further qualification	12.5%	14.4%	
Working part-/full-time, %	39.0%	38.3%	.65
Wealth quintile, %			.11
Lowest	8.9%	9.9%	
2nd	16.6%	17.0%	
3rd	23.2%	22.8%	
4th	25.1%	27.2%	
Highest	26.2%	23.2%	
Occupational status across lifespan, %			.62
Managerial/professional occupations	37.7%	38.4%	
Intermediate occupations	27.7%	27.7%	
Routine/manual occupations	34.6%	33.9%

At baseline, when comparing baseline depression rates between infrequent and frequent cultural engagement, those who engaged frequently had a 24% lower odds of having depression (Table 4, analysis i). They also had a 14% lower odds of experiencing depression at any point over the following 12 years (Table 4, analysis ii). When repeating analyses using an alternative threshold (never vs any engagement) all results remained (see supplementary material).

**Table 4:** Odds ratios for experiencing depression over a 12 year period: results from propensity matching analyses [infrequent vs frequent cultural engagement].

	OR	95% CI	p
(i) Odds or having depression at baseline matched by age, gender and SES	0.76	0.64–0.90	**.001**
(ii) Odds of experiencing depression over 12 years matched by age, gender and SES	0.86	0.76–0.98	**.026**

### 3.3 Study 3

When using the entire sample again (Table 1) and applying fixed-effects models (which take account of all time-invariant characteristics included in Study 1, plus any other time-invariant characteristics that are unobserved), amongst individuals who engaged frequently cultural activities there was a 38% lower odds of experiencing depression compared to those who never engaged (Table 5 analysis i). These results were similar (33% lower odds) when considering if some aspects of SES were in fact time-varying (Table 5 analysis ii). When repeating analyses using an alternative threshold (never vs any engagement) all results remained (see supplementary material).

**Table 5:** Odds ratios for experiencing depression over a 12 year period: results from fixed effects analyses [infrequent vs frequent cultural engagement].

	OR	95% CI	p
(i) Odds of experiencing depression when culturally engaged (n = 5,752^a^)	0.62	0.57–0.67	**<.001**
(ii) Odds of experiencing depression, assuming wealth is time-varying (n = 5752^a^)	0.67	0.62–0.73	**<.001**

a N smaller as only individuals who vary are included in the analysis.

## 4 Discussion

This paper confirms previous reports that cultural engagement is linked with a lower odds of depression amongst adults aged 50 and above by demonstrating a robust association in a nationally-representative sample of older adults. It further investigated whether SES is the explanatory factor for this relationship using three different statistical approaches. While SES does explain around half of the association between cultural engagement and depression, we found no evidence that it either acts as a moderator, or the main explanatory factor, with independent associations maintained across all three statistical approaches.

A key strength of this paper was that we used statistical triangulation, integrating results from three different statistical approaches to explore the relationship between cultural engagement, SES and depression (Lawlor et al., 2016). This meant that while each individual test has specific sources of potential bias, many of these sources are unrelated to each other. For example, logistic regression models condition on confounders but residual imbalance between groups can still bias results. However, we were able to confirm that our results were not biased by this limitation by also applying propensity matching, which ensures that both groups are comparable. Similarly, logistic regression models operate under the assumption that there is no un-measured confounding, which is challenging when exploring SES as it is a complex construct to measure. However, as SES is often relatively stable in older age, we were able to confirm that our results were also not biased by this limitation by applying fixed effects regression models, which take account of all stable confounders, even if they are unmeasured. Further, while logistic regression and propensity matching explore cultural engagement at just one point in time, fixed effects models including the time-varying nature of cultural engagement. All of our approaches provided consistent results, strengthening our confidence in concluding that SES is not the sole explanation for the associations between cultural engagement and mental health. There are, though, some further limitations that should be mentioned. First, all three studies used observational data, and therefore a causal link between cultural engagement and mental health cannot be assumed. Additionally, we used self-reports of depressive symptoms through a validated measure to categorise people as having experienced depression or not. However, this categorisation did not involve a clinical diagnosis. Third, we were only able to explore cultural engagement but not active arts participation due to suitable data not being available within ELSA. This therefore remains to be considered in future studies.

In concluding, SES does explain some of the longitudinal association between cultural engagement and mental health but it is not an overall explanation for the association. Nor does there appear to be a different relationship between cultural engagement and mental health amongst those of different levels of SES. This suggests that although there is strong literature showing a social gradient in cultural engagement (Alderson et al., 2007; Reeves and Vries, 2016), this relationship does not obscure an additional relationship between cultural engagement and mental health. However, the fact that higher SES is associated with more frequent engagement and the fact that it explains nearly half of the association between cultural engagement and mental health indicates that, in population terms, SES is still an important determinant of the salutogenic impact of culture. It is notable that a number of studies have suggested that not only is there a social gradient in cultural engagement but also that cultural engagement may contribute to health capital, including how individual interact with health services and their health literacy (Kobayashi et al., 2015; Shim, 2010). Consequently, differences in cultural engagement across different levels of SES could be contributing to inequalities in mental health. As economic and cultural inequalities have been found to be reciprocal (Warde et al., 2009), developing programmes that engage individuals in cultural activities could help to remedy this problem.

## Funding

The English Longitudinal Study of Ageing was developed by a team of researchers based at the University College London, NatCen Social Research, the Institute for Fiscal Studies and the University of Manchester. The data were collected by NatCen Social Research. The funding is provided by National Institute of Aging Grant R01AG017644 and a consortium of UK government departments coordinated by the Economic and Social Research Council. DF is supported by the Wellcome Trust [205407/Z/16/Z] and the MARCH Mental Health Network funded by the Cross-Disciplinary Mental Health Network Plus initiative supported by UK Research and Innovation [ES/S002588/1].
