# Supplementary material for: Cultural engagement and mental health: Does socio-economic status explain the association?
Source: Soc Sci Med. 2019 Sep;236:112425. doi: 10.1016/j.socscimed.2019.112425 (PMC6695288; doi:10.1016/j.socscimed.2019.112425)
Supplement: Multimedia component 1 [file mmc1.docx]

Supplementary Material

**Supplementary Table 1: Demographic and socio-economic factors by frequency of cultural engagement for all respondents [no cultural engagement vs any cultural engagement]**

|  | Unmatched data (Study 1 & Study 3)  N=8,780 | |  |
| --- | --- | --- | --- |
|  | No cultural engagement (N=1,605) | Any cultural engagement (N=7,175) | p |
| Age, mean (SD) | 70.1 (10.7) | 66.2 (9.8) | **<.001** |
| Female, % | 52.2% | 55.7% | **.01** |
| Education, % |  |  | **<.001** |
| No qualification | 69.0% | 38.7% |  |
| Qualification at age 16 / GCE / O level | 9.9% | 18.2% |  |
| Qualification at age 18 / A level | 18.7% | 29.0% |  |
| Degree / further qualification | 2.5% | 14.2% |  |
| Working part-/full-time, % | 18.3% | 34.7% | **<.001** |
| Wealth quintile, % |  |  | **<.001** |
| Lowest | 37.0% | 16.2% |  |
| 2^nd^ | 24.9% | 18.9% |  |
| 3^rd^ | 18.6% | 20.4% |  |
| 4^th^ | 12.7% | 21.6% |  |
| Highest | 6.8% | 23.0% |  |
| Occupational status across lifespan, % |  |  | **<.001** |
| Managerial/professional occupations | 16.4% | 34.1% |  |
| Intermediate occupations | 20.0% | 25.8% |  |
| Routine/manual occupations | 63.6% | 40.1% |  |

**Supplementary Table 2: Odds ratios for experiencing depression over a 12 year period: results from logistic regression analyses [no cultural engagement vs any cultural engagement]**

|  | OR | 95% CI | p |
| --- | --- | --- | --- |
| (i) Odds of experiencing depression over 12 years adjusted only for age and gender | 0.43 | 0.38-0.48 | **<.001** |
| (ii) Odds of experiencing depression over 12 years adjusted for age, gender and SES | 0.64 | 0.56-0.73 | **<.001** |
| (iii) Odds of experiencing depression over 12 years adjusted for age, gender, SES & baseline depression | 0.79 | 0.68-0.93 | **.004** |
| (iv) Adjusted odds of developing depression over 12 years if free from depression at baseline | 0.74 | 0.62-0.88 | **<.001** |
| (v) Odds of experiencing depression over 12 years stratified by SES |  |  |  |
| Lowest two wealth quintiles (n=3,497) | 0.67 | 0.56-0.81 | **<.001** |
| Highest three wealth quintiles (n=5,265) | 0.56 | 0.45-0.68 | **<.001** |
| No educational qualifications (m=3,880) | 0.72 | 0.61-0.86 | **<.001** |
| Educational qualifications at age 16 or above (n=4,893) | 0.50 | 0.40-0.62 | **<.001** |
| Routine/manual occupational status (n=3,894) | 0.72 | 0.60-0.86 | **<.001** |
| Intermediate or managerial/professional occupation status (n=4,857) | 0.54 | 0.43-0.66 | **<.001** |

**Supplementary Table 3: Demographic and socio-economic factors by frequency of cultural engagement for matched pairs of respondents [no cultural engagement vs any cultural engagement]**

|  | Matched data (Study 2)  (N=2,654) | |  |
| --- | --- | --- | --- |
|  | No cultural engagement (N=1,327) | Any cultural engagement (N=1,327) | p |
| Age, mean (SD) | 68.8 (10.1) | 68.8 (9.5) | .85 |
| Female, % | 54.3% | 53.4% | .62 |
| Education, % |  |  | .58 |
| No qualification | 64.4.4% | 62.5% |  |
| Qualification at age 16 / GCE / O level | 10.9% | 16.4% |  |
| Qualification at age 18 / A level | 21.8% | 18.2% |  |
| Degree / further qualification | 2.9% | 2.9% |  |
| Working part-/full-time, % | 21.4% | 18.2% | **.02** |
| Lowest wealth quintile, % | 29.8% | 28.9% | .44 |
| Occupational status across lifespan, % |  |  | .59 |
| Managerial/professional occupations | 18.2% | 17.6% |  |
| Intermediate occupations | 19.6% | 22.3% |  |
| Routine/manual occupations | 62.2% | 60.1% |  |

**Supplementary Figure 1: Percentage bias across demographics and SES before and after matching [no cultural engagement vs any cultural engagement]**


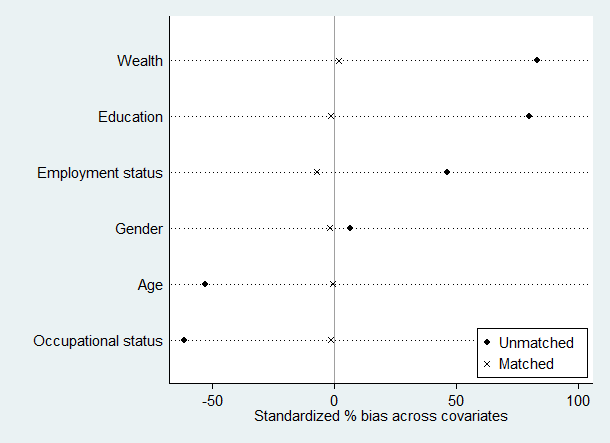


**Supplementary Table 4: Odds ratios for experiencing depression over a 12 year period: results from propensity matching analyses [no cultural engagement vs any cultural engagement]**

|  | OR | 95% CI | p |
| --- | --- | --- | --- |
| (i) Odds or having depression at baseline matched by age, gender and SES | 0.60 | 0.50-0.71 | **<.001** |
| (ii) Odds of experiencing depression over 12 years matched by age, gender and SES | 0.70 | 0.60-0.82 | **<.001** |

**Supplementary Table 5: Odds ratios for experiencing depression over a 12 year period: results from fixed effects analyses [no cultural engagement vs any cultural engagement]**

|  | OR | 95% CI | p |
| --- | --- | --- | --- |
| (i) Odds of experiencing depression when culturally engaged (n=5,752^a^) | 0.60 | 0.55-0.64 | **<.001** |
| (ii) Odds of experiencing depression, assuming wealth is time-varying (n=5,752 ^a^) | 0.65 | 0.61-0.70 | **<.001** |
| ^a^ N smaller as only individuals who vary are included in the analysis | | | |
